# Reproductive skew, fighting costs and winner–loser effects in social dominance evolution

**DOI:** 10.1111/1365-2656.13691

**Published:** 2022-03-29

**Authors:** Olof Leimar, Redouan Bshary

**Affiliations:** ^1^ Department of Zoology Stockholm University Stockholm Sweden; ^2^ Institute of Biology University of Neuchâtel Neuchâtel Switzerland

**Keywords:** aggression, distribution of reproductive success, evolutionary game theory, opt‐out loser effect, reinforcement learning, social hierarchy

## Abstract

1. Social hierarchies are often found in group‐living animals and can be formed through pairwise aggressive interactions. The dominance rank can influence reproductive success (RS) with a skew towards high‐ranking individuals.
2. Using game theory, we investigate how the opportunity for differently ranked individuals to achieve RS influences the costs of hierarchy formation and the strength of winner and loser effects.
3. In our model, individuals adjust their aggressive and submissive behaviour towards others through reinforcement learning. The learning is based on rewards and penalties, which depend on relative fighting ability. From individual‐based simulations, we determine evolutionary equilibria of traits such as learning rates. We examine situations that differ in the extent of monopolisation of contested RS by dominants and in the proportion of total RS that is contested.
4. The model implements two kinds of fighting costs: a decrease in effective fighting ability from damage (loss of condition) and a risk of mortality that increases with the total accumulated damage. Either of these costs can limit the amount of fighting.
5. We find that individuals form stable dominance hierarchies, with a positive correlation between dominance position and fighting ability. The accumulated costs differ between dominance positions, with the highest costs paid by low or intermediately ranked individuals. Costs tend to be higher in high‐skew situations.
6. We identify a ‘stay‐in, opt‐out’ syndrome, comprising a range from weaker (stay‐in) to stronger (opt‐out) winner–loser effects. We interpret the opt‐out phenotype to be favoured by selection on lower ranked individuals to opt out of contests over social dominance, because it is more pronounced when more of the total RS is uncontested.
7. We discuss our results in relation to field and experimental observations and argue that there is a need for empirical investigation of the behaviour and reproductive success of lower ranked individuals.

## Graphical abstract

Using a game theory model of social hierarchy formation, this work presents novel results on how the amount of fighting, and fighting damage, vary with rank position. The authors identify new kinds of winner–loser effects, referred to as a ‘stay‐in, opt‐out syndrome’.

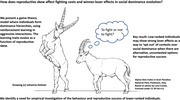

## 1 INTRODUCTION

Social hierarchies often influence the distribution of reproductive success (RS) in group‐living animals, with a skew towards higher success for dominant individuals (Clutton‐Brock, 1998; Clutton‐Brock & Huchard, 2013; Ellis, 1995; Strauss et al., 2022). The mating systems where dominance interactions can allocate RS might extend beyond those of a group of individuals equally utilising an area, to also include systems with a spatial structure, such as leks and some forms of territoriality. To gain a broader perspective on empirical studies of such systems, and to inspire further investigation, it is of interest to derive theoretical predictions about how the relation between an individual's dominance rank and its RS affects the amount of fighting and the costs of forming a dominance hierarchy, as well as such things as winner and loser effects.

Recent game theory models of social dominance (Leimar, 2021; McNamara & Leimar, 2020) have used learning about differences in fighting ability as a behavioural mechanism that can give rise to within‐sex dominance hierarchies, through pairwise interactions with aggressive and submissive behaviours. In these models, learning is implemented as actor‐critic learning, which is a commonly used form of reinforcement learning (Sutton & Barto, 2018). Individuals have genetically determined traits that function as parameters for the learning mechanism and can evolve to adapt learning to different situations. As discussed by Leimar (2021), work in neuroscience provides support for the idea that social dominance relations develop through processes that are similar to reinforcement learning (Dwortz et al., 2022; Kumaran et al., 2016; Ligneul et al., 2016; Qu et al., 2017; Zhou et al., 2018).

Here we extend the previous models of hierarchy formation to examine how reproductive skew influences fighting costs and winner and loser effects. First, we compare situations that differ in the proportion of total RS that is contested (i.e. is allocated based on rank), ranging from all of RS to a small part. We also examine different degrees of concentration of contested RS to higher ranks, from all of contested RS going to the top rank to a linear relation between rank and contested RS. Second, we introduce two types of costs of fighting damage: a loss in condition and vigour from damage, reducing an individual's effective fighting ability (as suggessted by Parker, 1974), and an increased risk of mortality from damage, with mortality eliminating RS. In comparison, in Leimar (2021) all of RS was contested, with a linear relation between rank and RS, and costs and benefits were decrements and increments to payoffs, without any specific interpretation (as is often the case in game theory). For simplicity, we assume an annual life cycle, with a single reproductive season. Our main aim is to provide predictions on how the amount and cost of fighting depend on the rank position, for different kinds of distributions of RS over ranks, and to relate this to winner–loser effects.

Our analysis applies to group‐living animals, but could also apply to situations with nearby territories or nesting sites, or display arenas on a lek, that differ in how valuable they are for reproduction and that are allocated according to a dominance hierarchy. The model might represent groups of males with contested mating opportunities, or females with contested foraging opportunities or nesting sites. Individuals are unrelated in the model, so it could apply to the dispersing sex in species where one sex disperses and the other is philopatric, and to either sex if both sexes disperse.

Among the examples of factors that can influence how male RS is distributed over ranks are difficulties for high‐ranking males to control matings in a group with several other active males, as in red junglefowl (McDonald et al., 2017), the synchrony of receptivity of females in a group, with higher synchrony reducing the possibilities for high‐ranking males to monopolise matings, as has been found in primate species (Ostner et al., 2008), and alternative tactics that allow lower ranked males to achieve matings, as described in the Alpine ibex (Willisch & Neuhaus, 2009). For females, the quality distribution of nesting sites and foraging opportunities can similarly shape the distribution of RS over ranks (Collias et al., 1994). Our assumptions about the distribution of RS are meant to capture such effects in a stylised manner. Uncontested RS in our model could in practice correspond to sources of RS separate from and unrelated to dominance interactions, but could also represent the RS obtained by the lowest rank, for instance a low‐quality display arena on a lek.

Based on what is known from previous game theory models of social dominance, as well as from the long‐standing study of single, pairwise contests, one would predict that the life‐history costs of fighting should be higher when a greater proportion of lifetime RS depends on winning dominance interactions. For our model, this corresponds to finding higher costs when a greater proportion of RS is contested and when that proportion is more skewed towards the top ranks. It is less clear how costs should depend on rank; there is no previous evolutionary analysis of this question. We examine if costs are higher for low‐, medium‐ or for high‐ranked individuals, and how this depends on the distribution of RS.

In our model, winner and loser effects are consequences of an individual's tendency to generalise the learning from winning or losing against an opponent to other, new opponents, in the same way as in Leimar (2021). Winner and loser effects have been investigated experimentally (Hsu et al., 2006; Rutte et al., 2006), but so far it is not known which circumstances favour their evolution. Here we explore the hypothesis that loser effects could be a way for lower ranked individuals to limit their involvement in contests over dominance, thus lowering their costs. This ought to be favoured when the lower ranks can gain uncontested RS and, additionally, contested RS is heavily skewed towards high‐ranking individuals. We refer to this possibility as an opt‐out loser effect and describe it as one end of a stay‐in, opt‐out syndrome.

In the following, we briefly describe our model, present a number of results from individual‐based evolutionary simulations and illustrative results from hypothetical winner–loser effect experiments. We discuss the implications of our results on costs of dominance interactions and winner–loser effects in relation to observations of reproductive skew in social hierarchies. We also discuss how our model could be changed to take into account such things as multi‐year life histories and overlapping generations.

## 2 MATERIALS AND METHODS

### 2.1 The model

**FIGURE 1:**
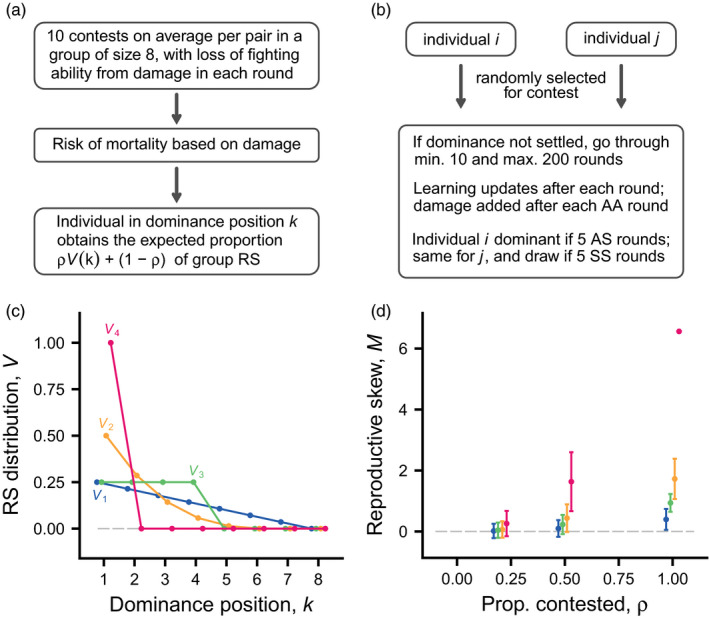
Elements of the model. (a) During a season there is a sequence of contests, with loss of effective fighting ability ${\hat{q}}_{i}$ from damage in each fighting round, followed by mortality risk and reproduction. The total expected reproductive success (RS) of a group is 16 (on average one daughter and one son per individual). A proportion (ρ) of the RS is contested, and the remaining proportion (1−ρ) is shared equally. Four distributions of contested RS over ranks k are studied, where k=1 is top ranked. They are denoted $V_{1}$, $V_{2}$, $V_{3}$ and $V_{4}$. Panel (b) summarises a contest for a randomly selected pair of group members. Panels (c) and (d) illustrate the distributions of RS. (c) The curves $V_{1},V_{2},V_{3},V_{4}$ (colour coded) show the different shapes of distributions of contested RS used in simulations. For each curve, the sum of the RS values is 1. (d) Mean (±*SD*) of the multinomial reproductive skew index M, computed over 10,000 replicates of a group of size 8 that produces a total of 16 offspring (mean RS of 2 per group member), for different RS distributions. The skew values are shown as functions of the proportion (ρ) of RS that is contested, for different distributions of contested RS, colour coded as in (c)

As mentioned, our model is an extension of a previous one (Leimar, 2021), with a new implementation of how dominance interactions occur over the season and how fitness benefits (RS) and costs (loss of condition and mortality) come about. In the previous model, interactions in a group consisted of a sequence of rounds, each with randomly selected opponents, and fitness effects were represented as increments to payoffs (benefits and costs) that were translated into reproduction at the end of interactions. In the current model, interactions are structured into multi‐round contests, which might better correspond to natural interactions. Fitness effects are given a concrete life‐history representation, with benefits as acquired RS, such as mating, and costs as decreases in effective fighting ability and mortality from fighting damage. Figure 1 and Table 1 give an overview of these aspects.

**TABLE 1:** Definitions and notation for the model

Notation	Definition or explanation
RS	$\text{Reproductive success}\;\left(\text{number of offspring}\right)$
ρ	Proportion of totalRSthat is contested
$V\left(k\right)$	$\text{Contested}\;\mathrm{RS}\;\text{proportion going to}\;\mathrm{rank}\;k\left(k=1\;\text{highest}\right)$
$V_{1},V_{2},V_{3},V_{4}$	$\text{Different shapes of distribution}\;V\left(k\right)$
M	$\text{Reproductive skew index from Ross}\;\mathrm{et}\;\mathrm{al}.\left(2020\right)$
$q_{i}$	$\text{Quality}\;\left(\text{fighting ability}\right)\;\text{of individual}\;i$
$\mu_{q},\sigma_{q}$	$\text{Mean and}\;\mathrm{SD}\;\text{of}\;\left(\text{normal}\right)\;\text{distribution of quality};\mu_{q}=0$
${\hat{q}}_{\mathrm{it}}$	Damage‐adjusted quality of individuali
A,S	Available actions:Ais aggressive,Sis submissive
$D_{\mathrm{it}}$	Accumulated fighting damage foriupto timet;eachAA $\text{round between}\;i\;\text{and}\;j\;\text{increases damage}\;\mathrm{by}\;e^{-\left({\hat{q}}_{\mathrm{it}}-{\hat{q}}_{\mathrm{jt}}\right)}$
$c_{0}$	$\text{Parameter for loss of}\;\left(\text{adjusted}\right)\;\text{quality cost}:{\hat{q}}_{\mathrm{it}}=q_{i}-c_{0}D_{\mathrm{it}}$
$c_{1}$	$\text{Parameter for mortality cost};\text{survival is}\;e^{-c_{1}D_{\mathrm{it}}}$
$h_{\mathrm{iit}}=f_{i}\theta_{\mathrm{iit}}$	Generalised component of preference for actionAattimet
$f_{i},\theta_{\mathrm{iit}}$	Degree of generalisation and learned weight for individuali
$h_{\mathrm{ijt}}=\left(1-f_{i}\right)\theta_{\mathrm{ijt}}$	Opponent specific component of preference forAattimet
$\theta_{\mathrm{ijt}}$	$\text{Learned weight in opponent}‐\text{specific component}\;h_{\mathrm{ijt}}$
$\theta_{0i}$	$\text{Starting value of}\;\theta_{\mathrm{iit}}\;\text{and}\;\theta_{\mathrm{ijt}}$
$\xi_{\mathrm{ijt}}$	$\begin{array}{c} \text{Observation}\;\mathrm{by}\;i,\text{meeting}\;j\;\mathrm{at}\;\text{time}\;t,\text{of relative quality} \end{array}$
$a_{0},\varepsilon_{\mathrm{ijt}}$	$\text{Weights}\;\mathrm{on}\;q_{i},q_{j},\text{and random error in observation}\;\xi_{\mathrm{ijt}}$
σ	$\mathrm{SD}\;\text{of}\;\left(\text{normally distributed}\right)\;\text{random error}\;\varepsilon_{\mathrm{ijt}}$
$\gamma_{0i}$	$\text{Slope parameter for}\;i\;\text{in preference component}\;\gamma_{0i}\xi_{\mathrm{ijt}}$
$p_{\mathrm{ijt}}$	Probability touseactionAbyiwhen meetingjattimet
$l_{\mathrm{ijt}}$	$\text{logit of}\;p_{\mathrm{ijt}},\text{referred to}\;\mathrm{as}\;\text{the preference for the action}\;A,$ $\text{defined}\;\mathrm{as}\;l_{\mathrm{ijt}}=h_{\mathrm{iit}}+h_{\mathrm{ijt}}+\gamma_{0i}\xi_{\mathrm{ijt}}$
${\hat{v}}_{\mathrm{ijt}}$	$\text{Estimated value}\;\left(\text{reward}\right)\;\mathrm{by}\;i\;\text{when meeting}\;j\;\mathrm{at}\;\text{time}\;t$
$w_{\mathrm{iit}},w_{\mathrm{ijt}}$	$\text{Generalised and opponent}‐\text{specific learned weights in}\;{\hat{v}}_{\mathrm{ijt}}$
$w_{0i}$	$\text{Starting value of}\;w_{\mathrm{iit}}\;\text{and}\;w_{\mathrm{ijt}}$
$g_{0i}$	$\begin{array}{c} \text{Slope parameter in}\;{\hat{v}}_{\mathrm{ijt}}=f_{i}w_{\mathrm{iit}}+\left(1-f_{i}\right)w_{\mathrm{ijt}}+g_{0i}\xi_{\mathrm{ijt}} \end{array}$
$R_{\mathrm{ijt}}$	Perceived rewardbyiwhen meetingjattimet
$v_{i}$	$\begin{array}{c} \text{Perceived reward}\;\mathrm{by}\;i\;\text{of performing the aggressive action}\;A \end{array}$
$e_{\mathrm{ijt}},\sigma_{p}$	$\text{Random influence}\;e_{\mathrm{ijt}}\;\text{with}\;\mathrm{SD}\;\sigma_{p}\;\text{in penalty from}\;\mathrm{AA}\;\text{round}$ $\begin{array}{c} \text{between}\;i\;\text{and}\;j,\text{given}\;\mathrm{by}\;\exp\left(-{\hat{q}}_{\mathrm{it}}+{\hat{q}}_{\mathrm{jt}}+e_{\mathrm{ijt}}\right) \end{array}$
$\alpha_{\mathrm{\theta i}},\alpha_{\mathrm{wi}}$	$\text{Learning rates for updates}\;\mathrm{by}\;i\;\text{of weights in}\;l_{\mathrm{ijt}}\;\text{and}\;{\hat{v}}_{\mathrm{ijt}}$
$\beta_{i}$	$\text{Bystander learning rate},\text{similar to}\;\alpha_{\mathrm{\theta i}}$

Individuals meet in pairwise contests over dominance, with several contests per group member (Figure 1a), giving opportunities for group members to form a dominance hierarchy. For instance, a pair with similar fighting abilities can have a long contest, or several contests with fighting (which happened very rarely in our simulations), potentially settling which of them dominates the other. A contest (Figure 1b) can be thought of as an opportunity for a dominance interaction; if dominance is already settled, there is no interaction. If there is an interaction, the model assumes a minimum and maximum number of rounds, to ensure that group members have experience of interacting with each other. A contest ends if there is a specified number of successive rounds with either a clear direction, so that one individual is aggressive and the other submits, which then indicates dominance, or a specified number of rounds where both submit, which indicates a draw. This aspect of the model is inspired by how dominance is often scored in experiments on hierarchy formation. The sequence of contests can produce a linear hierarchy, but it is also possible that there are cycles, or that some dominance relations remain undetermined, for instance if some group members avoid being aggressive towards each other, or if some continue fighting.

The probability of survival from the contests to reproduction depends on an individual's total accumulated damage (Figure 1a). Each round of fighting adds to damage, in a way that depends on the relative fighting abilities of the interacting individuals.

For individuals that survive, the RS (e.g. matings) is distributed according to rank (Figure 1c). If some, or even all, ranks are undetermined at this stage, those ranks are randomly assigned (so if all individuals keep fighting, refusing to submit, RS is randomly assigned; we used the score‐structure method from Landau (1951) to assign ranks, see SI). We investigate four distributions of contested RS over the ranks k (Figure 1c). They differ in how strongly the top ranks in a hierarchy monopolise RS. The model allows for uncontested RS, which is distributed to all (surviving) group members, irrespective of contest outcomes; the proportion of total RS that is contested is denoted ρ. The amount of reproductive skew that would result from these assumptions about acquired RS, for a hypothetical case where there is a linear dominance hierarchy, is shown in Figure 1d. To describe reproductive skew, we use the recently developed multinomial index (Ross et al., 2020).

Important concepts and notation for the model are summarised in Table 1. A detailed model description, including those aspects that are the same as in the previous model (Leimar, 2021), is presented in the Supporting Information.

The work did not involve experiments or other empirical observations, so no ethical approval is needed.

### 2.2 Evolutionary simulations

Individuals are assumed to have genetically determined traits. The evolution of the traits is studied in individual‐based simulations. The traits for individual i are (Table 1): degree of generalisation, $f_{i}$; preference and value learning rates, $\alpha_{\mathrm{\theta i}}$, $\alpha_{\mathrm{wi}}$; bystander learning rate $\beta_{i}$; initial preference for action A, $\theta_{0i}$; initial estimated value, $w_{0i}$; effect of observations on preference and value functions, $\gamma_{0i}$, $g_{0i}$; and perceived reward from performing A, $v_{i}$.

In evolutionary simulations, each trait is determined by an unlinked diploid locus with additive alleles. Alleles mutate with a probability of 0.002 per generation, with normally distributed mutational increments. The standard deviation of mutational increments for each trait was adjusted to correspond to the range of trait variation between cases (Table S1), to ensure that simulations could locate evolutionary equilibria.

A simulated population consisted of 2,000 groups of eight individuals taking part in dominance interactions (either males or females), plus eight individuals of the other sex, resulting in a total population size of N=32,000. Each interacting individual was assigned a quality $q_{i}$, independently drawn from a normal distribution with mean zero and standard deviation $\sigma_{q}$.

Offspring for the next generation were formed by randomly selecting parents in a group for each of 16 offspring from that group, with probabilities proportional to an individual's expected RS for the sex involved in interactions and uniformly for the other sex. The offspring were randomly dispersed over the groups in the next season, to eliminate any effects of relatedness in local groups. For each case reported in Table S1, simulations were performed over intervals of 5,000 generations, repeated at least 100 times, to estimate mean and standard deviation of traits at an evolutionary equilibrium.

#### 2.2.1 Standard parameter values

The following ‘standard values’ of parameters (Table 1) were used: cost as loss of condition (loss of effective fighting ability) from damage, $c_{0}=0.02$; mortality cost from damage, $c_{1}=0.0004$; distribution of individual quality (fighting ability), $\sigma_{q}=0.50$; observations of relative quality, $a_{0}=0.707$, σ=0.50; and perceived penalty variation, $\sigma_{p}=0.25$. For these parameter values, around 50% of the variation in the observations $\xi_{\mathrm{ijt}}$ by individuals in each round is due to variation in relative fighting ability, $q_{i}-q_{j}$ (this means that an individual obtains substantial, but not complete, information about relative fighting ability from the observation $\xi_{\mathrm{ijt}}$).

## 3 RESULTS

### 3.1 Dominance hierarchy formation

**FIGURE 2:**
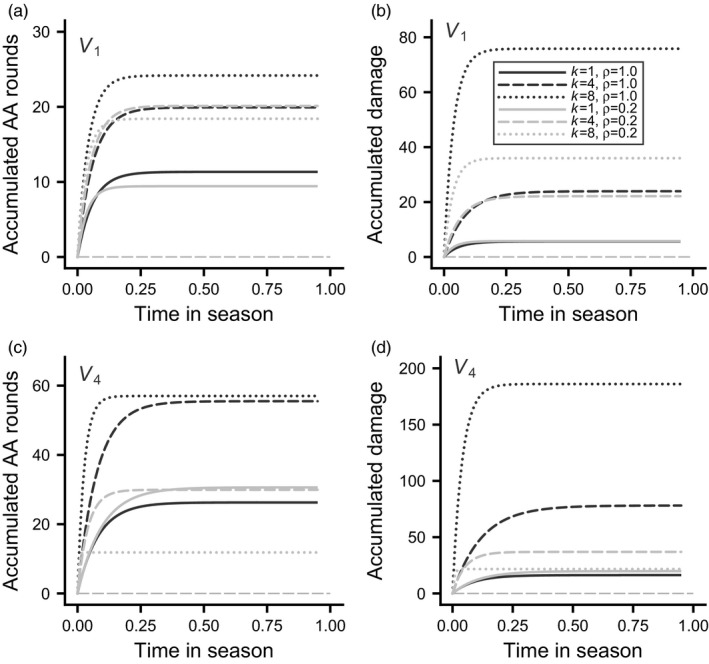
Examples of fitted curves for accumulated number of AA rounds (fighting rounds) and accumulated damage as functions of time in the season, for different dominance positions k. The cases 1, 3, 10 and 13 in Table S1 (with proportions contested RS of ρ=1.0,0.2 and distributions $V_{1}$ and $V_{4}$) are shown. The curves for different ranks k (top, middle and bottom) are respectively bold, dashed and dotted, and the value of ρ is indicated by dark/light grey, as shown in the legend in panel (b). The learning parameters are given by the mean values in Table S1. For each case, 2,000 groups of eight individuals were simulated. (a) Accumulated AA rounds as a function of time in the season, for different ranks k and values of the proportion contested RS (ρ) when the distribution of contested RS is given by $V_{1}$ in Figure 1c. (b) Accumulated fighting damage for the situation in (a). Panels (c) and (d) show the same as (a) and (b), but for the distribution $V_{4}$ in Figure 1c. Time on the x‐axes has been defined such that 1.0 corresponds to completion of all contests. Note that the scales differ between the y‐axes

Using four distributions of contested RS over dominance ranks ($V_{1},V_{2},V_{3},V_{4}$, Figure 1c) in combination with three values of the proportion contested RS (ρ=0.2,0.5,1.0), we analysed 12 cases of individual‐based evolutionary simulations (summarised in Table S1). In all these cases, interactions lead to the formation of a dominance hierarchy. The course of aggressive interactions over the season is illustrated in Figure 2 for the cases with distributions $V_{1}$ and $V_{4}$ and proportions ρ=0.2 and 1.0 of contested RS (these include the extremes of the range of cases), with curves for top‐, middle‐ and bottom‐ranked individuals. Time in the season is defined such that contests start at t=0 and end at t=1, at which time there has been an opportunity for 10 contests per pair. As can be seen, most of the fighting occurs early (Figure 2a,c), and this is when most damage is incurred (Figure 2b,d). The explanation is that there are more and longer fights early in the season. In the overwhelming majority of groups and cases, pairs of individuals settle their dominance relation already in their first contest. There are illustrations of the contests in a single group in Figures S1 and S2. Figure S2 shows that fighting rounds (AA rounds) tend to occur in the early contests, and for some of the later contests there is no fighting, only displays, with one contestant being submissive and the other aggressive.

### 3.2 Distribution of fighting and damage over ranks

**FIGURE 3:**
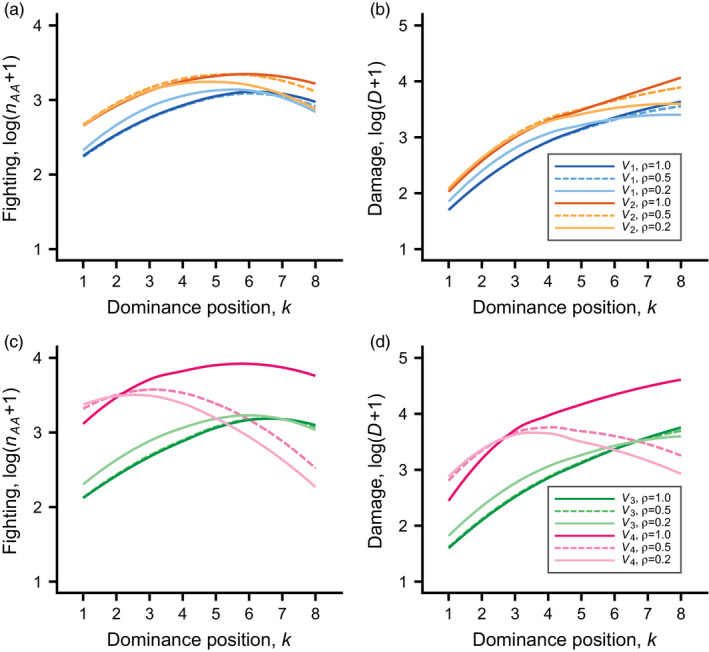
Fitted curves (loess fits) for log‐transformed total number of AA rounds (fighting rounds) and total damage as functions of dominance position k, for the 12 evolutionary simulations in Table S1 (the learning parameters are given by the mean values in the table), with cases 1 to 6 in (a) and (b), and cases 7 to 12 in (c) and (d). For each case, 2,000 groups of eight individuals were simulated. The legends in panels (b) and (d) indicate the different cases, with colour coding as in Figure 1c. See Figure S4 and S5 for illustration of individual data points and their distributions

For the distribution $V_{1}$, with a linear dependence of contested RS on rank, bottom‐ranked individuals go through more fighting rounds and accumulate more damage than top ranks when all RS is contested (ρ=1.0; Figures 2a,b and 3a,b). The effect is more extreme for damage than for fighting, because lower ranks tend to have lower fighting ability and also to lose more condition from fighting stronger opponents. The effects are similar but less extreme when most RS is uncontested (ρ=0.2; Figures 2a,b and 3a,b). For the distribution $V_{4}$, with the highest skew of contested RS, there is overall more fighting and damage (Figures 2c,d and 3c,d), but the dependence on rank is different, in particular when most RS is uncontested (ρ=0.2), in which case bottom‐ranked individuals fight less than the top‐ranked, thus reducing their damage from fighting. This is a manifestation of the opt‐out loser effect.

Figure 3 shows the total number of fighting rounds and the total damage as functions of rank for all the 12 cases in Table S1. For each shape of the distribution of contested RS (colour coded), the overall pattern is that top‐ranked individuals (k=1) fight more and bottom‐ranked (k=8) less when there is more uncontested RS (smaller ρ, Figure 3a,c). In particular for the distribution $V_{4}$, for which only the top rank obtains contested RS, the bottom‐ranked individuals fight considerably less when there is more uncontested RS (Figure 3c). As mentioned, this is a manifestation of the opt‐out loser effect. In some cases, lower ranked individuals do not fight at all against certain of the higher ranked opponents. This happens when an individual is submissive already in the first contest with an opponent. The phenomenon is more prevalent when lower ranked individuals have little to gain by fighting (Figure S3), and is thus related to the opt‐out loser effect.

It should be noted that there is much variation between the groups, depending on such things as the particular fighting abilities $q_{i}$ in a group and randomness in contest outcomes (Figures S1, S2, S4 and S5 illustrate some of the variation). In general, in presenting results we show statistical model fits (nonlinear regressions, including loess regressions), to ease comparison between the cases.

### 3.3 Relation between rank and fighting ability

The rank that an individual obtains in its group is related to its fighting ability $q_{i}$, although the correlation is not perfect. For the cases shown in Figure 3, the mean over groups of the correlation between the rank k (or rather, −k) and the fighting ability $q_{i}$ range from 0.85 to 0.89, except for the cases with $V_{4}$, where they are slightly lower, ranging from 0.75 to 0.80. The explanation is that when only the top‐ranked individual obtains contested RS, which holds for the $V_{4}$ distribution, the rankings among the lower ranked individuals in a group matter less and are therefore less sharply determined by relative fighting ability. For comparison, we computed Elo ratings (see SI), which are often used to measure rank in social hierarchies (Albers & de Vries, 2001; Neumann et al., 2011). The correlations between an individual's rank and its Elo rating tended to be higher than that between rank and fighting ability, with means ranging from 0.90 and 0.95 for the different cases.

### 3.4 Winner and loser effects

**FIGURE 4:**
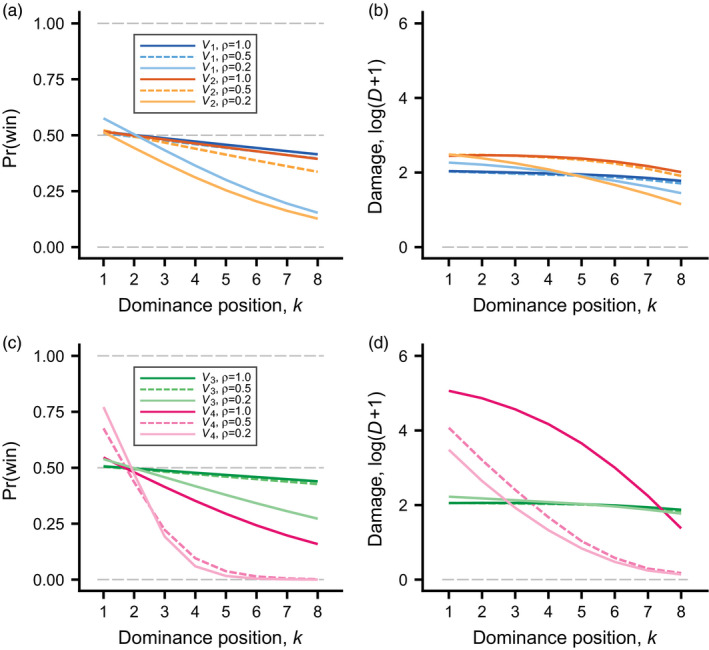
Illustration of hypothetical winner–loser effect experiments. Each group member that survived over the season had a staged interaction with a matched (equal fighting ability, $q_{i}=q_{j}$) new and naive opponent. A group member was assumed to have recovered from previous fighting damage, but to remember its own previous learning. A staged pair had up to 10 contests, each as described in Figure 1b, ending when dominance was settled. The different cases (colour coded) are those in Figure 3 (and in Table S1). For each case, there were 2,000 simulated groups, including winner–loser experiments. (a) and (c) Fitted (logistic regression) probability of winning (becoming dominant) for a group member interacting with a matched, naive opponent, as a function of the group member's previous dominance position. (b) and (d) Fitted (loess fits) log‐transformed damage D from contests with the matched opponent. For matched opponents, the damage is equal to the number of fighting rounds

To examine winner and loser effects for the different cases, we simulated experiments where group members who survived over the season met new, matched opponents in staged contests. We assumed that group members remembered and generalised their previous learning, which is what gives rise to winner and loser effects in the model, but we allowed them to recover from any loss of condition from the fights in the group. As can be seen in Figure 4a,c, there are winner effects for the top ranks, and noticeably stronger loser effects for lower ranks, with the strongest effects occurring when most RS was uncontested (ρ=0.2), illustrating the opt‐out loser effect. The total damage against a matched opponent (Figure 4b,d) shows a contrasting pattern from the total damage in Figure 3, with the top ranks taking the most damage. This shows that top‐ranked individuals are prepared to fight hard when they meet a matched opponent. The case $V_{4},\rho =1.0$ is the most extreme in terms of reproductive skew and has more damage for high ranks than in the other cases (Figure 4d).

The differences between the cases in Figures 2, 3, 4 are consequences of variation in several of the evolved learning traits between the cases (Table S1). For winner–loser effects, the generalisation factor $f_{i}$ is the most important of the traits (as was found by Leimar, 2021), and this is illustrated in Figure S6 for the case of $V_{1},\rho =1$. Increasing generalisation from its evolved value to a higher one ($f_{i}=0.5$) leads to stronger winner–loser effects, such that the top ranks fight more and the bottom ranks fight less (Figure S6a).

### 3.5 Different costs of fighting

For the case of $V_{1},\rho =1$, we investigated some of the non‐evolutionary consequences of having loss of condition as one of the costs of fighting. Eliminating that cost ($c_{0}=0$) caused most ranks to fight somewhat more (Figure S6a).

We also examined the evolutionary consequences of eliminating the loss‐of‐condition cost, letting the risk of mortality be the only cost of fighting. Table S2 shows the outcome of evolutionary simulations with $c_{0}=0$ and $c_{1}=0.002$ (see Table 1 for explanation of parameters). The resulting patterns of fighting and damage over the ranks are shown in Figure S7, which can be compared with Figure 3. A main difference is that there tends to be more fighting overall. Examining the effect of the proportion of contested RS (ρ), for a given shape of the distribution contested RS, there is less fighting for lower values of ρ for all ranks (Figure S7). A likely explanation is that the higher mortality risks (compare Tables S1 and S2) caused top‐ranked individuals to be more cautious (lower ranks became even more cautious), in order to avoid losing all potential RS.

Finally, we investigated the consequences of eliminating all costs from our model, with the expectation that strategies of refusing to submit should be favoured. From simulations (data not shown), we found that without either loss‐of‐condition or mortality costs ($c_{0}=0,c_{1}=0$), dominance hierarchies do not form because individuals keep fighting and RS becomes uncorrelated with fighting ability.

## 4 DISCUSSION

We found that learning traits evolved to values such that dominance hierarchies were quickly formed, early in the season (Figure 2). Typically, a pair of individuals settled their relative dominance already in their first contest, and most of the fighting and damage occurred in the early fights. The distribution of RS over the ranks of a hierarchy strongly influenced the evolution of learning traits (Table S1) and, as a consequence, also influenced the costs of competing for dominance. Costs were higher when more of the RS was contested and when contested RS was more skewed towards the top ranks (Figures 2 and 3), which is in accordance with our predictions.

The dependence of the number of fighting rounds and the fighting damage on rank position, and the way these patterns differ between the cases (Figures 2 and 3), represent a new type of model results for social dominance. In the situations we examined, individuals of an intermediate rank (i.e. neither top nor bottom ranked) fought most during hierarchy formation (Figure 3), although in many cases bottom‐ranked individuals accumulated most fighting damage (Figure 3). An exception was when contested RS was heavily skewed towards the top ranks ($V_{4}$ distribution), and low‐ranked individuals could gain RS by opting out of fighting (i.e. ρ=0.2,0.5; Figure 3).

The results of our hypothetical winner–loser effect experiment (Figure 4) are consistent with and to some extent explain these patterns of fighting and damage over ranks. We found winner effects for the top ranks and considerably stronger loser effects for lower ranked individuals (Figure 4), with a particularly strong loser effect for highly skewed contested RS in combination with RS available for lower ranks ($V_{4}$, ρ=0.2,0.5; Figure 4c). These conditions are the most favourable for the opt‐out loser effect. This can be contrasted with the linear dependence of contested RS on rank ($V_{1}$ distribution), where lower ranked individuals instead stay in the competition for contested RS and loser effects are considerably weaker (Figure 4a). Note here that we use the terms stay‐in and opt‐out to refer to the behaviour of lower ranked individuals, to describe whether they fully participate in competition for contested RS or relatively quickly opt out.

Comparing with the results by Leimar (2021) on winner–loser effects, the strongest loser effects we found here are clearly stronger, in terms of the evolved values of the generalisation factor $f_{i}$ (Table S1). The likely explanation is that the model by Leimar (2021) assumed a linear dependence on rank of the reproductive benefits. Our modelling here has thus identified a new kind of explanation for winner–loser effects, that potentially could be tested empirically, for instance by comparing species with different relations between rank and RS. The variation in behaviour of lower ranks we found, including the stay‐in, opt‐out syndrome, could also be studied empirically, by comparing how sharply dominance positions among the lower ranks are formed relative to those among the higher ranks, and if this varies between species.

Examples of issues previous modelling has examined include whether winner and loser effects can emerge and potentially explain social dominance without there being differences in fighting ability (van Doorn, Hengeveld, & Weissing, 2003; van Doorn, Weissing, & Hengeveld, 2003), and what an assumption of either winner or loser effects might mean for the structure of dominance hierarchies (Dugatkin, 1997). Previous work has, however, left open the question of when such effects are expected to evolve (Mesterton‐Gibbons et al., 2016).

A finding by Leimar (2021) is that very strong winner–loser effects can be detrimental to the formation of dominance hierarchies, in particular in larger groups (hierarchies form more slowly or, in extreme cases, fail to form). While this is correct we argue here that, in situations of highly skewed contested RS in combination with opportunities for uncontested RS, strong loser effects can be adaptive and have the function of limiting fighting costs for weaker individuals. These individuals have little to gain and more to lose by persisting in contests for dominance. A consequence of such loser effects can be that the top ranks of a hierarchy go to the strongest fighters, but for lower ranks the hierarchy becomes less sharply defined. It could well be that hierarchies with diffuse lower ranks are commonly occurring, but the issue needs further empirical work. It is also of interest to study which pairs of individuals avoid fighting. In a comparative study, McDonald and Shizuka (2013) found that datasets often contained non‐interacting dyads. In our model, this might correspond to dyads that do not fight. Based on our results (Figure S3), one would predict that this is more common when the opting‐out loser effect is strong, and that it is caused by lower ranked individuals avoiding aggressive interactions with higher ranks.

Obtaining data on both lifetime RS and social dominance is of course challenging, but there are several studies. There is strong support for a general reproductive advantage of higher rank, but genetic data on ungulates, pinnipeds and primates also show that monopolisation of mating by dominant males in polygynous mammals is typically not complete (Alberts et al., 2006; Coltman et al., 1999; Coltman et al., 2002; Hoffman et al., 2003; Hogg & Forbes, 1997; Pemberton et al., 1992; Pörschmann et al., 2010; Stopher et al., 2011; Twiss et al., 2006; Worthington Wilmer et al., 1999; Wroblewski et al., 2009). In order to test our model predictions, more information on the lower ranked individuals would, however, be needed.

An essential ingredient in our model is the assumed loss‐of‐condition cost, and thus loss of effective fighting ability, from fighting damage. The idea that an important cost of escalated fighting might be loss of fighting ability was introduced by Parker (1974), in the context of single contests. Such costs have been fairly little explored in previous game theory modelling, but empirically oriented work shows that they are likely to be important (e.g. Briffa & Lane, 2017; Briffa & Sneddon, 2007; Emberts & Wiens, 2021; Lane & Briffa, 2017). They include physiological effects, such as exhaustion, as well as reductions in fighting skill, and various kinds of injury and damage to weaponry. There are studies illustrating the consequences of exhaustion and damage when individuals are involved in several contests in succession, such as during hierarchy formation (e.g. Clutton‐Brock et al., 1979; Clutton‐Brock & Albon, 1979; Geist, 1966). These consequences include a turnover of top‐ranked individuals as the season progresses. It would be of interest to examine in greater detail how such loss‐of‐condition costs vary with dominance positions.

As a comparison, we performed evolutionary simulations with risk of mortality from damage as the only cost of fighting (Table S2; Figure S7). A notable difference is a much higher mortality for this alternative, which might well be higher than what is typically observed in fieldwork on social dominance (e.g. Wilkinson & Shank, 1976). This suggests that costs in the form of loss of condition could be an important explanation for relatively low risks of mortality in hierarchy formation even if reproductive skew is high. Another interesting difference is that, when the costs are mainly in the form of loss of condition, individuals of high fighting ability, ending up in the top ranks, accumulate relatively little cost in comparison with weaker and lower ranked individuals. The reason is that loss‐of‐condition costs are particularly problematic for weak individuals. These individuals accumulate such costs more quickly, causing an even higher rate of cost accumulation, potentially leading to a ‘cost explosion’. Avoiding such accelerating costs could be a reason for weaker individuals to limit their fighting.

Finally, concerning modelling styles, we note that the game theory approach we use here investigates the evolution of traits that control specific behavioural mechanisms, such as the parameters of reinforcement learning, over a range of situations. This produces relatively complex models that need individual‐based simulations for evolutionary analysis, but the approach has the distinct advantage that it can incorporate biologically realistic elements, such as variation in fighting ability, learning processes and different costs of fighting. The models can also give predictions about variability in behaviour, both within and between social groups.

Our current model could be extended to include elements like multi‐year life histories, territoriality or relatedness between group members. Among the ingredients needed for this to succeed are reasonable specifications of traits and perceptions of the interacting individuals, such as effects of age on aggressiveness and whether individuals distinguish relatives from non‐relatives. We believe such endeavours benefit from collaboration between modellers, experimentalists and biologists with experience from the field, because this helps overcoming the considerable challenges of linking theoretical constructs to natural situations.

## CONFLICT OF INTEREST

The authors declare no conflict of interest.

## AUTHORS' CONTRIBUTIONS

O.L. and R.B. designed the model; O.L. implemented and performed model analysis; O.L. wrote the manuscript; R.B. helped revise the manuscript.

## Supporting information


Supinfo

## Data Availability

No data were analysed in this work. C++ source code for the individual‐based simulations is available at GitHub, as well as from Zenodo (Leimar, 2022), together with instructions for compilation on a Linux operating system https://github.com/oleimar/socdom3 Zenodo https://doi.org/10.5281/zenodo.6361417.
