# Supplementary material for: Reproductive skew, fighting costs and winner–loser effects in social dominance evolution
Source: J Anim Ecol. 2022 Mar 29;91(5):1036–46. doi: 10.1111/1365-2656.13691 (PMC9315160; doi:10.1111/1365-2656.13691)
Supplement: Supplementary file 1 — Supinfo [file JANE-91-1036-s001.pdf]

Supporting information for “Reproductive skew, fighting costs, and winner-loser effects in social-dominance evolution”

Olof Leimar[1, *] and Redouan Bshary[2]

[1]Department of Zoology, Stockholm University, SE-106 91 Stockholm, Sweden
[2]Institute of Biology, University of Neuchâtel, Neuchâtel, Switzerland
[*]Corresponding author; e-mail: olof.leimar@zoologi.su.se

Statistical fitting of curves to simulated data

Fitted curves are shown in Figs 2 to 4 and S4 to S7. Non-linear regressions from the R statistical package (version 4.0.5; `https://www.R-project.org/`) were used, with the aim of giving a reasonable visual summary of the individual data points from simulations. The variation around fitted curves can be judged from Figs S4 and S5.

We used either the `nls` function, (Fig. 2), the `loess` function (Figs 3, 4b, 4d, S4, S5, S6, S7), or logistic regression using the `glm` function (Figs 4a, 4c). For the `nls` fits of number of fighting rounds and damage vs. time t we used $A\big(1 - \exp(-Bt)\big)$ as the non-linear function with parameters A and B to be fitted.

Model details

Brief descriptions of model elements appear in Table 1. Several elements of the model are similar to those in a previous one (Leimar 2021). These are the observations in a round, including assumptions about individual recognition, the actions A and S, the action preferences and estimated values, the implementation of action exploration, and the learning updates, including bystander learning.

Observations and actions

The model simplifies a round of interaction into two stages. In the first stage, interacting individuals make an observation. Thus, individuals observe some aspect ξ of relative fighting ability and also observe the opponent's identity. The observation by an individual is statistically related to the difference in fighting ability between itself and the opponent, $q_i - q_j$. For the interaction between individuals i and j at time t, the observation is

$$\xi_{ijt} = a_0(q_i - q_j) + \epsilon_{ijt}, \tag{S1}$$

where $a_0 > 0$ and ϵ_{ijt} is an error of observation, assumed to be normal with mean zero and SD σ. Note here that the observations ξ_{ijt} refer to the original fighting abilities q_i and q_j, and not the effective fighting abilities (see below).

By adjusting the parameters σ_q, which is the SD of the distribution of q_i, and a_0 and σ from equation (S1), one can make the information about relative quality more or less accurate. The observation (ξ_{ij}, j) is followed by a second stage, where individual i chooses an action, and similarly for individual j. The model simplifies to only two actions, A and S, corresponding to aggressive and submissive behaviour.

Action preferences and estimated values

For an individual i interacting with j at time t, l_{ijt} denotes the preference for A. The probability that i uses A is then

$$p_{ijt} = \frac{1}{1 + \exp(-l_{ijt})}, \tag{S2}$$

so that the preference l_{ijt} is the logit of the probability of using A. The model uses a linear (intercept and slope) representation of the effect of ξ_{ijt} on the preference, and expresses l_{ijt} as the sum of three components

$$l_{ijt} = h_{iit} + h_{ijt} + \gamma_{0i}\xi_{ijt} = f_i\theta_{iit} + (1 - f_i)\theta_{ijt} + \gamma_{0i}\xi_{ijt}. \tag{S3}$$

Here $h_{iit} = f_i\theta_{iit}$ is a contribution from generalisation of learning from all interactions, $h_{ijt} = (1 - f_i)\theta_{ijt}$ is a contribution specifically from learning from interactions with a particular opponent j, and $\gamma_{0i}\xi_{ijt}$ is a contribution from the current observation of relative fighting ability. Note that for $f_i = 0$ the learning about each opponent is a separate thing, with no generalisation between opponents, and for $f_i = 1$ the intercept component of the action preference is the same for all opponents, so that

effectively there is no individual recognition (although the observations ξ_{ijt} could still differ between opponents). One can similarly write the estimated value $\hat{v}_{ijt}$ of an interaction as a sum of three components:

$$\hat{v}_{ijt} = f_i w_{iit} + (1 - f_i) w_{ijt} + g_{0i} \xi_{ijt}. \tag{S4}$$

The actor-critic method updates θ_{iit}, θ_{ijt}, w_{iit}, and w_{ijt} in these expressions based on perceived rewards, whereas f_i, γ_{0i}, and g_{0i} are genetically determined.

Exploration in learning

For learning to be efficient over longer time spans there must be exploration (variation in actions), in order to discover beneficial actions. Learning algorithms, including the actor-critic method, might not provide sufficient exploration (Sutton and Barto 2018), because learning tends to respond to short-term rewards. In the model, exploration is implemented as follows: if the probability in equation (S2) is less than 0.01 or greater than 0.99, the actual choice probability is assumed to stay within these limits, i.e. is 0.01 or 0.99, respectively. In principle the degree of exploration could be genetically determined and evolve to an optimum value, but for simplicity this is not implemented in the model.

Fighting damage and effective fighting ability

A group member i accumulates damage D_{it} from fighting. D_{it} refers to accumulated damage up to (but not including) round t. As a consequence of the damage, the individual's effective fighting ability is reduced from the original q_i to

$$\hat{q}_{it} = q_i - c_0 D_{it}, \tag{S5}$$

where c_0 is a parameter. Following an AA round between i and j, there is an increment to D_{it}:

$$D_{i,t+1} = D_{it} + \exp\bigl(-(\hat{q}_i - \hat{q}_j)\bigr), \tag{S6}$$

and similarly for j. The effective fighting abilities also determine the perceived costs (see below), and in this way they influencing the learning.

Perceived rewards

An SS interaction is assumed to have zero rewards, $R_{ijt} = R_{jit} = 0$. For an AS interaction, the aggressive individual i perceives a reward $R_{ijt} = v_i$, which is genetically determined and can evolve. The perceived reward for the submissive individual j is zero, $R_{jit} = 0$, and vice versa for SA interactions. If both individuals use A, some form of costly interaction or fight occurs, with perceived costs (negative rewards or penalties) that are influenced by the effective fighting abilities of the two individuals. The perceived rewards of an AA interaction are assumed to be

$$\begin{aligned} R_{ijt} &= v_i - \exp(-\hat{q}_{it} + \hat{q}_{jt} + e_{ijt}) \\ R_{jit} &= v_j - \exp(-\hat{q}_{jt} + \hat{q}_{it} + e_{jit}), \end{aligned} \tag{S7}$$

where e_{ijt} is a normally distributed random influence on the perceived penalty, with mean zero and standard deviation σ_p, and similarly for e_{jit}.

Learning updates

In actor-critic learning, an individual updates its learning parameters based on the prediction error (TD error)

$$\delta_{ijt} = R_{ijt} - \hat{v}_{ijt}, \tag{S8}$$

which is the difference between the actual perceived reward R_{ijt} and the estimated value $\hat{v}_{ijt}$. The learning updates for the θ parameters are given by

$$\begin{aligned} \theta_{ii,t+1} &= \theta_{iit} + \alpha_{\theta i} f_i \zeta_{ijt} \delta_{ijt} \\ \theta_{ij,t+1} &= \theta_{ijt} + \alpha_{\theta i}(1 - f_i) \zeta_{ijt} \delta_{ijt}, \end{aligned} \tag{S9}$$

where

$$\zeta_{ijt} = \begin{cases} 1 - p_{ijt} & \text{if A was chosen} \\ -p_{ijt} & \text{if S was chosen} \end{cases} \tag{S10}$$

is referred to as a policy-gradient factor and $\alpha_{\theta i}$ is the preference learning rate for individual i. Note that ζ_{ijt} will be small if p_{ijt} is close to one and individual i performed action A, which slows down learning, with a corresponding slowing down if p_{ijt} is close

to zero and S is chosen. There are also learning updates for the w parameters given by

$$\begin{aligned} w_{ii,t+1} &= w_{iit} + \alpha_{wi}\, f_i \delta_{ijt} \\ w_{ij,t+1} &= w_{ijt} + \alpha_{wi}\, (1 - f_i)\delta_{ijt}, \end{aligned} \tag{S11}$$

where α_{wi} is the value learning rate for individual i.

The updates to the policy parameters θ can be described using derivatives of the logarithm of the probability of choosing an action with respect to the parameters. Using equation (S2), we obtain

$$\zeta_{ijt} = \begin{cases} \frac{d \log \Pr(\mathrm{A})}{d\, l_{ijt}} = 1 - p_{ijt} & \text{if A was chosen} \\ \frac{d \log \Pr(\mathrm{S})}{d\, l_{ijt}} = -p_{ijt} & \text{if S was chosen} \end{cases} \tag{S12}$$

for the derivative of the logarithm of the probability of choosing an action, A or S, with respect to the preference for A, which corresponds to equation (S10). From equation (S3) it follows that

$$\begin{aligned} \frac{\partial l_{ijt}}{\partial \theta_{iit}} &= f_i \\ \frac{\partial l_{ijt}}{\partial \theta_{ijt}} &= 1 - f_i \end{aligned} \tag{S13}$$

and this gives the learning updates of the θ parameters in equation (S9). The updates of the w parameters of the value function can also be described using derivatives. From equation (S4) it follows that

$$\begin{aligned} \frac{\partial \hat{v}_{ijt}}{\partial w_{iit}} &= f_i \\ \frac{\partial \hat{v}_{ijt}}{\partial w_{ijt}} &= 1 - f_i \end{aligned} \tag{S14}$$

and this gives the learning updates of the w parameters in equation (S11).

Bystander updates

As in Leimar (2021), bystander effects are modelled as observational learning. When there is a dominance interaction in a group, individuals other then the interacting pair i and j, for instance an individual k, can use the outcome to update the learning parameters. Assume that individual k only performs this updating if i and j end their interaction by using AS or SA (because there is no clear ‘winner’ in AA and

SS interactions, and bystanders do not perceive the costs of AA interactions). The probabilities for individuals i and j to use A are p_{ijt} and p_{jit}, from equation (S2). These are 'true' values and are not known by individual k. However, given that the outcome is either AS or SA, one readily derives that the logit of the probability that it is AS is

$$\operatorname{logit} \Pr(\mathrm{AS}|\mathrm{AS} \text{ or } \mathrm{SA}) = l_{ijt} - l_{jit}. \tag{S15}$$

From equation (S3) one can see that this involves various learning parameters for i and j. For bystander learning an assumption is needed about how an individual k represents this logit. A simple assumption is that k represents the logit as

$$\operatorname{logit} \Pr(\mathrm{AS}|\mathrm{AS} \text{ or } \mathrm{SA}) = -\theta_{kit} + \theta_{kjt}, \tag{S16}$$

which entails that k does not use any information about q_i or q_j. The assumption is reasonable in that a large θ_{kit} means that k behaves as if individual i is weak, and similarly for θ_{kjt}. Using the notation

$$P_{kijt} = \frac{1}{1 + \exp(\theta_{kit} - \theta_{kjt})}, \tag{S17}$$

the bystander updates by k is assumed to be

$$\begin{aligned} \theta_{ki,t+1} &= \theta_{kit} - (1 - P_{kijt})\beta_k \\ \theta_{kj,t+1} &= \theta_{kjt} + (1 - P_{kijt})\beta_k \end{aligned} \tag{S18}$$

if i wins (outcome is AS) and

$$\begin{aligned} \theta_{ki,t+1} &= \theta_{kit} + P_{kijt}\beta_k \\ \theta_{kj,t+1} &= \theta_{kjt} - P_{kijt}\beta_k \end{aligned} \tag{S19}$$

if j wins (outcome is SA). The parameter β_k is a measure of how salient or significant a bystander observation is for individual k, and this parameter is assumed to be genetically determined and can evolve. These bystander updates are similar to the direct-learning updates of the actor component of the actor-critic model and were used in Leimar (2021).

There is also the possibility that the salience for a bystander of a contest outcome is influenced by additional information the bystander might have, either from current or from previous observations. For instance, if the observations in equation (S1) are

about relative size, a bystander might have estimates ξ_{ki} and ξ_{kj} of its own size in relation to the contestants i and j. Instead of the bystander updates above we might then have

$$\begin{aligned} \theta_{ki,t+1} &= \theta_{kit} - (1 - P_{kijt})\beta_k \exp(-\xi_{ki}) \\ \theta_{kj,t+1} &= \theta_{kjt} + (1 - P_{kijt})\beta_k \exp(\xi_{kj}) \end{aligned} \tag{S20}$$

if i wins (outcome is AS) and

$$\begin{aligned} \theta_{ki,t+1} &= \theta_{kit} + P_{kijt}\beta_k \exp(\xi_{ki}) \\ \theta_{kj,t+1} &= \theta_{kjt} - P_{kijt}\beta_k \exp(-\xi_{kj}) \end{aligned} \tag{S21}$$

if j wins (outcome is SA). These updates entail that a bystander k pays particular attention to wins by an individual perceived to be bigger than itself, and to losses by an individual perceived to be smaller. We used these updates in our simulations.

Life-history and reproductive season

There is an annual life cycle with a single reproductive season. Dominance interactions occur in groups of size g_s, with $g_s = 8$ for the individual-based simulations in Table S1. The season starts with a sequence of contests. Each contest is between a randomly selected pair of group members and there are $5g_s(g_s - 1)$ contests, i.e., on average 10 contests per pair. An individual's survival from the contests to reproduction depends on the accumulated damage. As a result of the contests, a dominance hierarchy is formed, and surviving group members acquire reproductive success (RS) according to their ranks. The purpose of the scheme is to implement a combination of hierarchy formation, resource acquisition, and mortality over the season in a way that allows both fitness benefits and costs to influence trait evolution. In principle, similar results could be achieved by, for instance, implementing a risk of mortality after each contest, or even after each round of interaction.

Contests

If a dominance relation has already been established between contestants i and j, there is no interaction. If not, the contestants go through a number of rounds, at minimum 10 rounds and at maximum 200 rounds of interaction. If there are 5 successive rounds where i uses A and j uses S (5 AS rounds), the contest ends and i is considered dominant over j, and vice versa if there are 5 successive SA rounds. Further, the contest ends in

a draw if there are 5 successive SS rounds.

Mortality from fighting damage

An individual with accumulated damage D_{it} survives from contests to reproduction with probability

$$\exp\big(-c_1 D_{it}\big). \tag{S22}$$

Dominance ranking and reproductive success

The ranking is among surviving individuals and is based of how many other group members an individual dominates (this measure is referred to as a score structure by Landau (1951). If some individuals dominate the same number of other group members, their relative rank is randomly determined (this happened occasionally in our simulations). As an extreme example, if all individuals would use action S in the contests, there would be no real dominance hierarchy, because each would dominate zero other group members, and all ranks would be randomly determined (this never happened in our simulations).

Surviving group members acquire reproductive success. A local group, containing 8 interacting individuals (if all survive) and 8 of the other sex, produces an expected number of 16 offspring. For each offspring, one parent of the interacting sex is drawn from the group with a probability proportional to $\rho V(k) + (1 - \rho)$ (Fig. 1a), where k is the rank, and a parent of the other sex is randomly drawn. For instance, if a linear hierarchy has been established and all survive, an individual with rank k (with $k = 1$ the top rank) obtains an expected RS of $\rho V(k) + (1 - \rho)$. In the next generation, each offspring disperses to a random local group. In this way, interacting individuals are unrelated.

Elo rating

Several approaches to Elo ratings have been used, differing in such things as the zero point of the scale and the amount to change ratings after a ‘win’ by one individual over another, or after a ‘draw’. There is similarity between updates of Elo ratings and the updates of action preferences for actor-critic learning described above. Here, however, we use the Elo rating just as a conventional measure or index of dominance rank, without further interpretation of what the scores might mean. The possible usefulness of this measure needs instead to be investigated. From our results here, Elo ratings appear useful in providing a description of a dominance hierarchy.

Let E_{it} be the Elo rating of group member i at time t. Initially all have rating $E_{i0} = 0$. If a contest between i and j ends with i becoming dominant over j, E_{it} is incremented by

$$2.5(1 - P_{ijt}), \tag{S23}$$

where

$$P_{ijt} = \frac{1}{1 + \exp(-E_{it} + E_{jt})}. \tag{S24}$$

The Elo rating of j, E_{jt}, is decremented by the same amount. If the contest ends in a draw, E_{it} is decremented by

$$2.5(P_{ijt} - 0.5), \tag{S25}$$

and E_{jt} is incremented by this amount. It can help the interpretation to think of P_{ijt} as the probability, before the interaction, of the outcome ('win', 'loss', or 'draw'). This, however, is just an interpretation that helps explaining why Elo ratings are defined in a certain way. Dominance relations differ from wins and losses in a tournament, so it is not certain that Elo ratings are useful for predicting outcomes of dominance interactions. One can, of course, investigate the usefulness for each particular case.

Table S1: Reproductive parameters, mean survival, and trait values (mean ± SD over 100 simulations, each over 5000 generations) for 12 different cases of individual-based evolutionary simulations of social dominance interactions. The cost parameters for loss of fighting ability and mortality are $c_0 = 0.02$ and $c_1 = 0.0004$ ('standard values').

case	ρ	V	Surv.	f_i	$\alpha_{\theta i}$	α_{wi}	βi
1	1.0	V_1	0.983	0.028 ± 0.006	90.7 ± 18.6	0.052 ± 0.008	1.167 ± 0.128
2	0.5	V_1	0.984	0.041 ± 0.011	65.9 ± 12.3	0.055 ± 0.010	1.217 ± 0.167
3	0.2	V_1	0.988	0.157 ± 0.029	11.8 ± 1.2	0.055 ± 0.016	2.148 ± 0.273
4	1.0	V_2	0.976	0.029 ± 0.008	169.3 ± 22.1	0.059 ± 0.009	0.275 ± 0.095
5	0.5	V_2	0.980	0.050 ± 0.012	83.3 ± 9.2	0.060 ± 0.009	0.311 ± 0.084
6	0.2	V_2	0.982	0.115 ± 0.019	48.4 ± 8.2	0.068 ± 0.015	0.434 ± 0.150
7	1.0	V_3	0.983	0.020 ± 0.005	109.1 ± 18.7	0.060 ± 0.009	1.746 ± 0.213
8	0.5	V_3	0.983	0.031 ± 0.007	61.3 ± 9.9	0.057 ± 0.010	1.781 ± 0.203
9	0.2	V_3	0.987	0.110 ± 0.026	11.9 ± 1.5	0.048 ± 0.013	2.312 ± 0.246
10	1.0	V_4	0.955	0.127 ± 0.064	84.5 ± 22.7	0.083 ± 0.028	0.153 ± 0.103
11	0.5	V_4	0.978	0.362 ± 0.035	29.8 ± 7.9	0.041 ± 0.015	0.187 ± 0.088
12	0.2	V_4	0.984	0.457 ± 0.025	15.7 ± 1.8	0.036 ± 0.012	0.217 ± 0.080

table continued

case	θ_{0i}	w_{0i}	γ_{0i}	g_{0i}	v_i
1	4.17 ± 0.18	-0.04 ± 0.01	1.22 ± 0.17	0.06 ± 0.01	0.83 ± 0.01
2	3.95 ± 0.20	-0.06 ± 0.01	1.28 ± 0.25	0.07 ± 0.01	0.81 ± 0.01
3	2.36 ± 0.26	-0.05 ± 0.03	1.05 ± 0.20	0.11 ± 0.03	0.78 ± 0.02
4	4.78 ± 0.17	-0.01 ± 0.01	0.96 ± 0.20	0.04 ± 0.01	0.89 ± 0.01
5	4.36 ± 0.12	-0.03 ± 0.01	0.96 ± 0.18	0.05 ± 0.01	0.87 ± 0.01
6	4.00 ± 0.19	-0.06 ± 0.01	1.02 ± 0.21	0.08 ± 0.02	0.83 ± 0.02
7	4.33 ± 0.18	-0.04 ± 0.01	1.16 ± 0.21	0.05 ± 0.01	0.84 ± 0.01
8	3.94 ± 0.17	-0.07 ± 0.01	1.18 ± 0.20	0.07 ± 0.01	0.82 ± 0.01
9	2.49 ± 0.24	-0.06 ± 0.03	1.03 ± 0.23	0.10 ± 0.03	0.78 ± 0.02
10	4.72 ± 0.36	0.05 ± 0.04	0.57 ± 0.36	0.07 ± 0.03	1.02 ± 0.03
11	3.92 ± 0.27	-0.05 ± 0.03	0.66 ± 0.26	0.08 ± 0.03	0.92 ± 0.03
12	3.47 ± 0.26	-0.08 ± 0.04	0.86 ± 0.27	0.11 ± 0.02	0.84 ± 0.02

Table S2: Same as Table S1, but for cases with no cost of loss of fighting ability ($c_0 = 0$) and higher mortality cost ($c_1 = 0.002$). Reproductive parameters, mean survival, and trait values (mean ± SD over 100 simulations, each over 5000 generations) for 12 different cases of individual-based evolutionary simulations of social dominance interactions.

case	ρ	V	Surv.	f_i	$\alpha_{\theta i}$	α_{wi}	βi
13	1.0	V_1	0.860	0.051 ± 0.014	44.1 ± 5.2	0.028 ± 0.005	1.078 ± 0.152
14	0.5	V_1	0.907	0.078 ± 0.014	55.1 ± 9.7	0.032 ± 0.006	1.198 ± 0.156
15	0.2	V_1	0.943	0.151 ± 0.026	32.4 ± 8.6	0.042 ± 0.012	1.871 ± 0.334
16	1.0	V_2	0.775	0.072 ± 0.021	64.0 ± 18.0	0.033 ± 0.006	0.524 ± 0.169
17	0.5	V_2	0.860	0.147 ± 0.014	53.7 ± 5.3	0.034 ± 0.006	0.406 ± 0.126
18	0.2	V_2	0.918	0.208 ± 0.016	54.6 ± 9.5	0.043 ± 0.008	0.453 ± 0.161
19	1.0	V_3	0.813	0.035 ± 0.010	32.4 ± 5.1	0.026 ± 0.005	2.122 ± 0.293
20	0.5	V_3	0.885	0.062 ± 0.017	48.0 ± 7.1	0.027 ± 0.005	1.435 ± 0.190
21	0.2	V_3	0.933	0.112 ± 0.020	39.2 ± 8.0	0.035 ± 0.009	1.565 ± 0.254
22	1.0	V_4	0.649	0.284 ± 0.024	21.4 ± 5.4	0.033 ± 0.013	0.211 ± 0.195
23	0.5	V_4	0.835	0.492 ± 0.019	15.9 ± 1.5	0.018 ± 0.005	0.160 ± 0.091
24	0.2	V_4	0.912	0.600 ± 0.022	15.0 ± 1.1	0.013 ± 0.003	0.664 ± 0.219

table continued

case	θ_{0i}	w_{0i}	γ_{0i}	g_{0i}	v_i
13	4.71 ± 0.13	-0.04 ± 0.01	0.79 ± 0.18	0.05 ± 0.01	0.89 ± 0.01
14	4.81 ± 0.26	-0.06 ± 0.01	1.32 ± 0.32	0.07 ± 0.02	0.82 ± 0.02
15	4.56 ± 0.40	-0.13 ± 0.03	2.19 ± 0.55	0.10 ± 0.03	0.67 ± 0.04
16	5.17 ± 0.23	0.00 ± 0.01	0.57 ± 0.19	0.04 ± 0.01	0.96 ± 0.01
17	4.83 ± 0.14	-0.03 ± 0.01	0.76 ± 0.21	0.05 ± 0.01	0.91 ± 0.01
18	5.03 ± 0.28	-0.08 ± 0.02	1.63 ± 0.37	0.08 ± 0.02	0.79 ± 0.02
19	4.75 ± 0.19	-0.03 ± 0.01	0.68 ± 0.21	0.05 ± 0.01	0.91 ± 0.01
20	4.73 ± 0.20	-0.05 ± 0.01	1.12 ± 0.29	0.06 ± 0.01	0.86 ± 0.01
21	4.57 ± 0.28	-0.13 ± 0.02	1.79 ± 0.33	0.09 ± 0.02	0.73 ± 0.02
22	5.72 ± 0.37	0.01 ± 0.05	0.65 ± 0.39	0.05 ± 0.02	0.95 ± 0.01
23	3.88 ± 0.26	-0.04 ± 0.02	0.57 ± 0.26	0.06 ± 0.02	0.94 ± 0.01
24	2.38 ± 0.30	-0.07 ± 0.02	0.56 ± 0.17	0.07 ± 0.02	0.89 ± 0.01

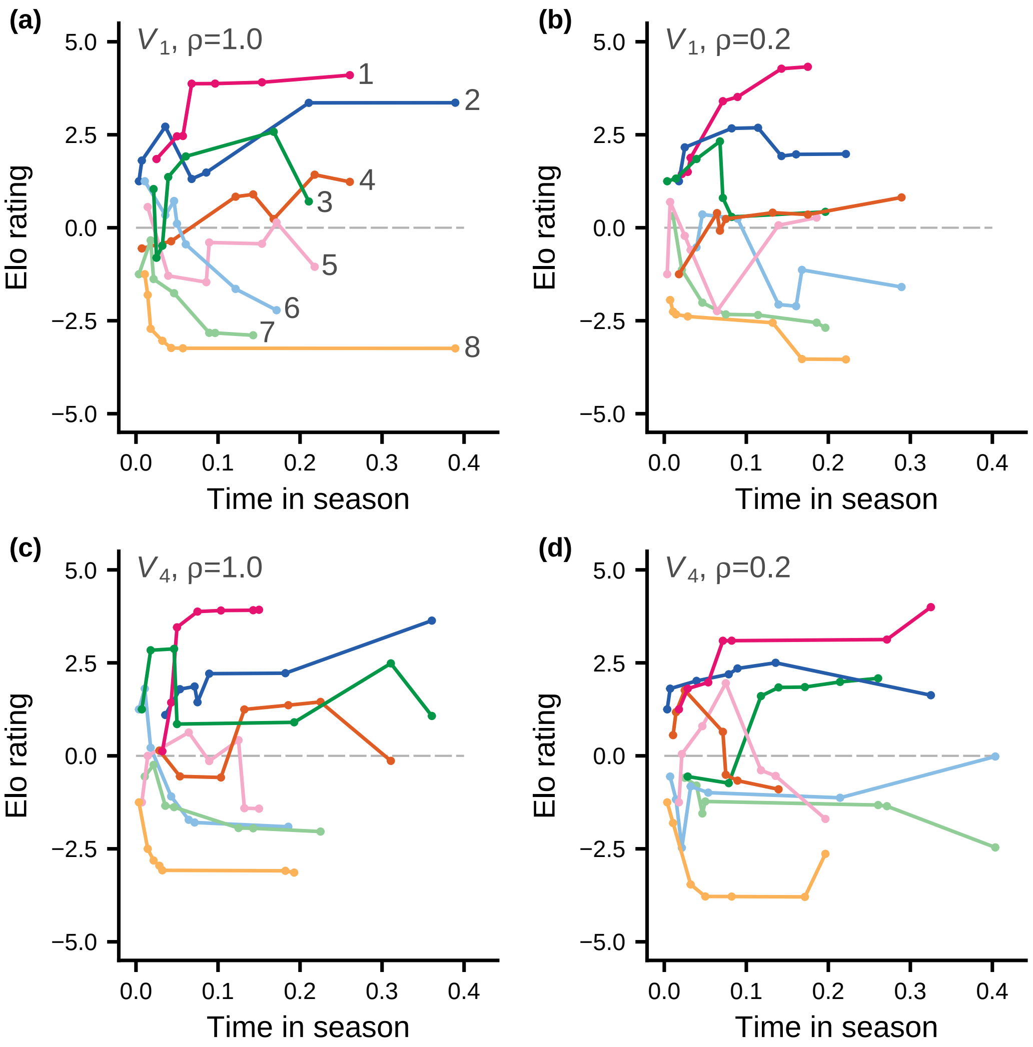

Figure S1: Elo ratings for individuals of different ranks as a function of time. Each panel shows one group for one of the cases illustrated in Fig. 2 (case 1, 3, 10, and 12 in Table S1, indicated by giving the shape of the distribution of contested RS and the proportion ρ of contested RS in each panel). The curves are colour coded according to an individual's rank, with labels in panel (a). Time is defined in the same way as in Fig. 2.

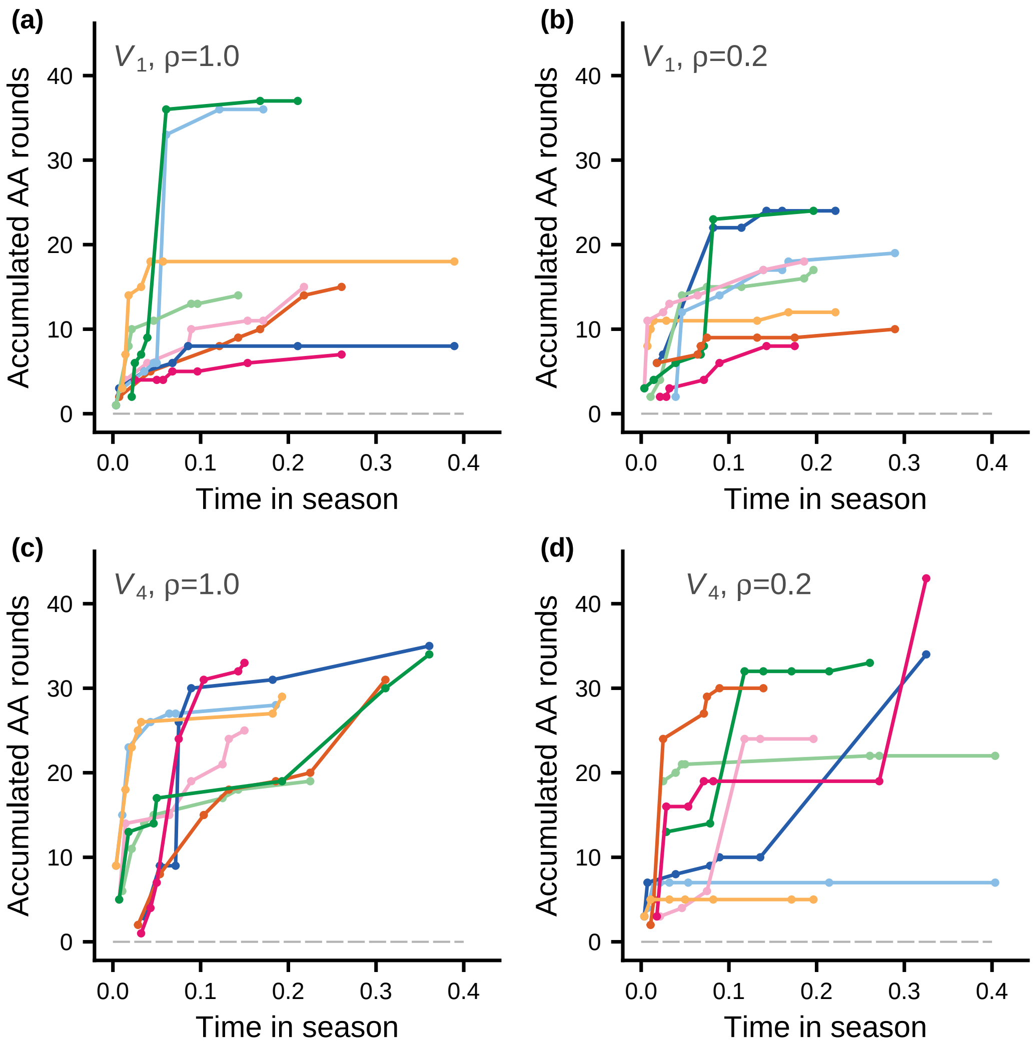

Figure S2: Accumulated number of AA rounds (fighting rounds) for individuals of different ranks as a function of time. The groups and cases and the colour coding of individual ranks are the same as in Fig. S1.

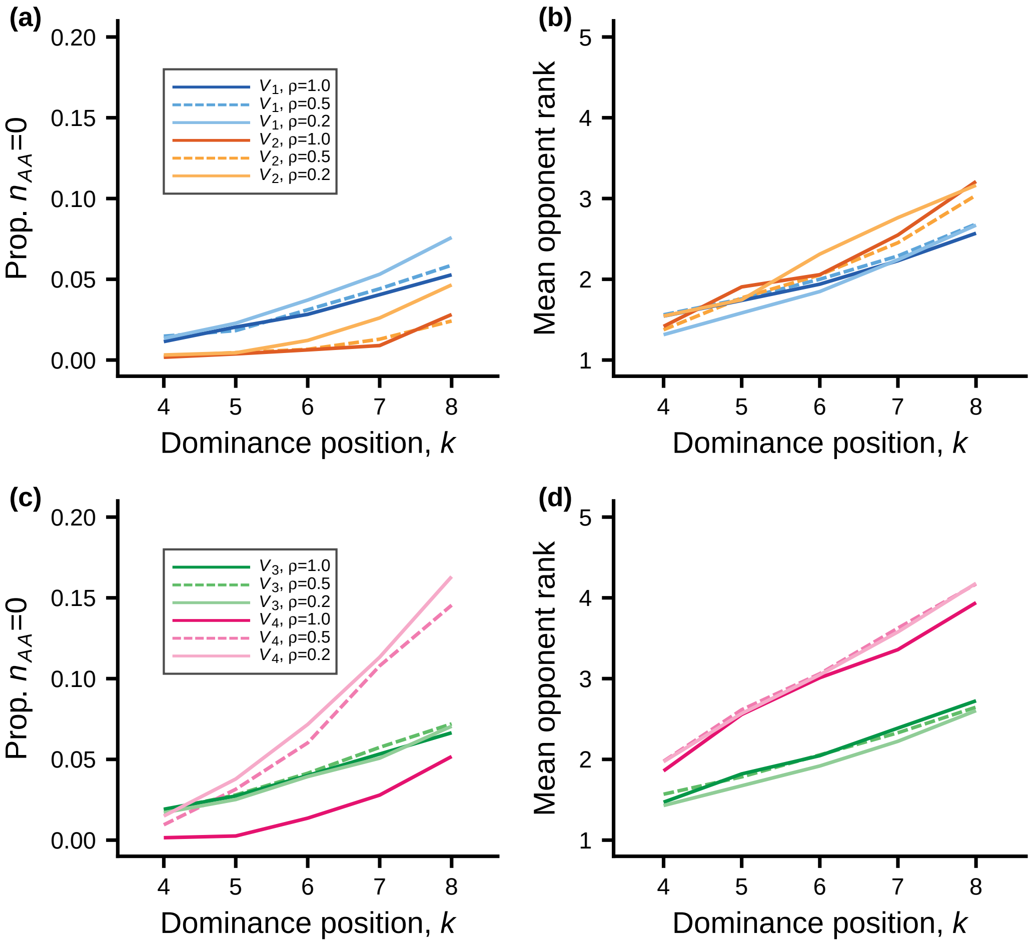

Figure S3: Illustration of contests where there is no fighting (number of AA rounds is zero), because the lower-ranked-to-be individual is submissive throughout the contest. Panels (a) and (c) show the proportion of all contests for individuals of a certain rank where the individual avoided fighting against a higher-ranked-to-be opponent, and panels (b) and (d) show the mean rank of this opponent. Only data for rank positions 4 to 8 are shown; higher-ranked-to-be individuals were rarely submissive throughout a contest. Colour coding and underlying simulation data are the same as in Fig. 3.

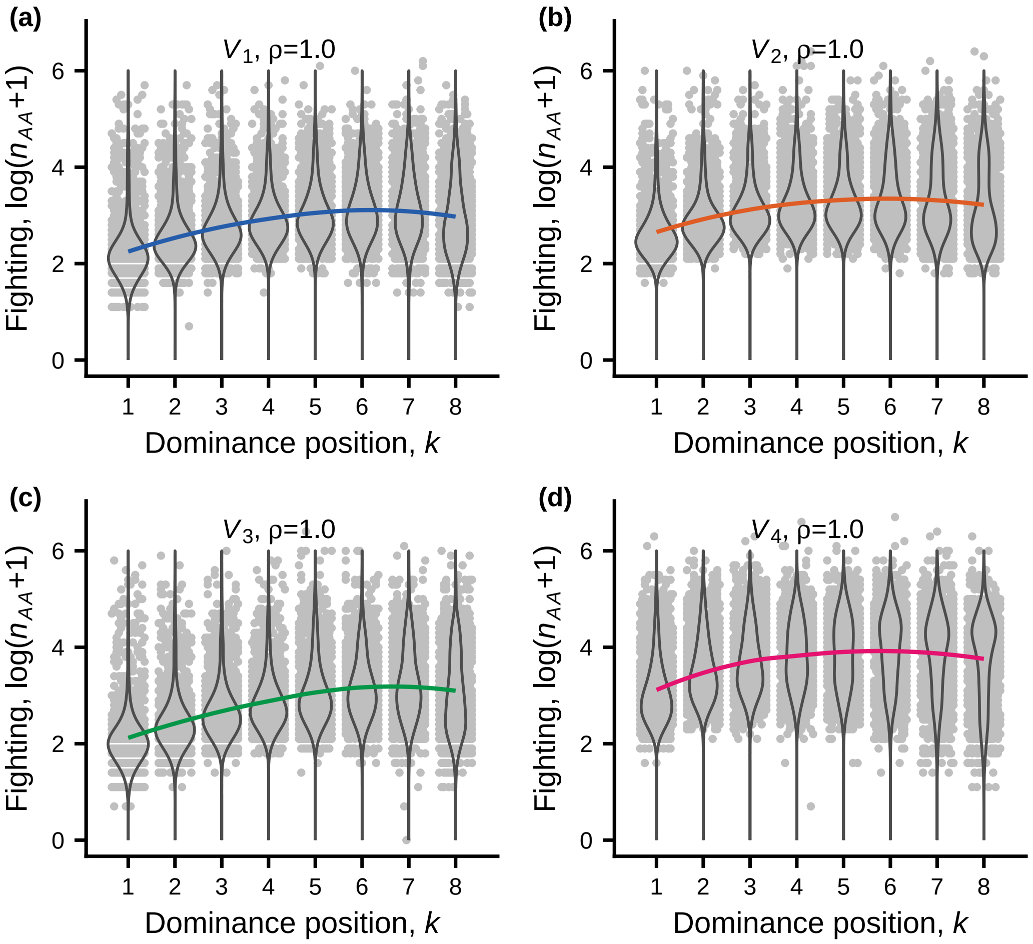

Figure S4: Simulated data (light grey points), density violins, and colour coded fitted curves of log-transformed total number of AA rounds (fighting rounds) for the cases in Fig. 3a, 3c with $\rho = 1$. The x-values of the points are jittered for visibility.

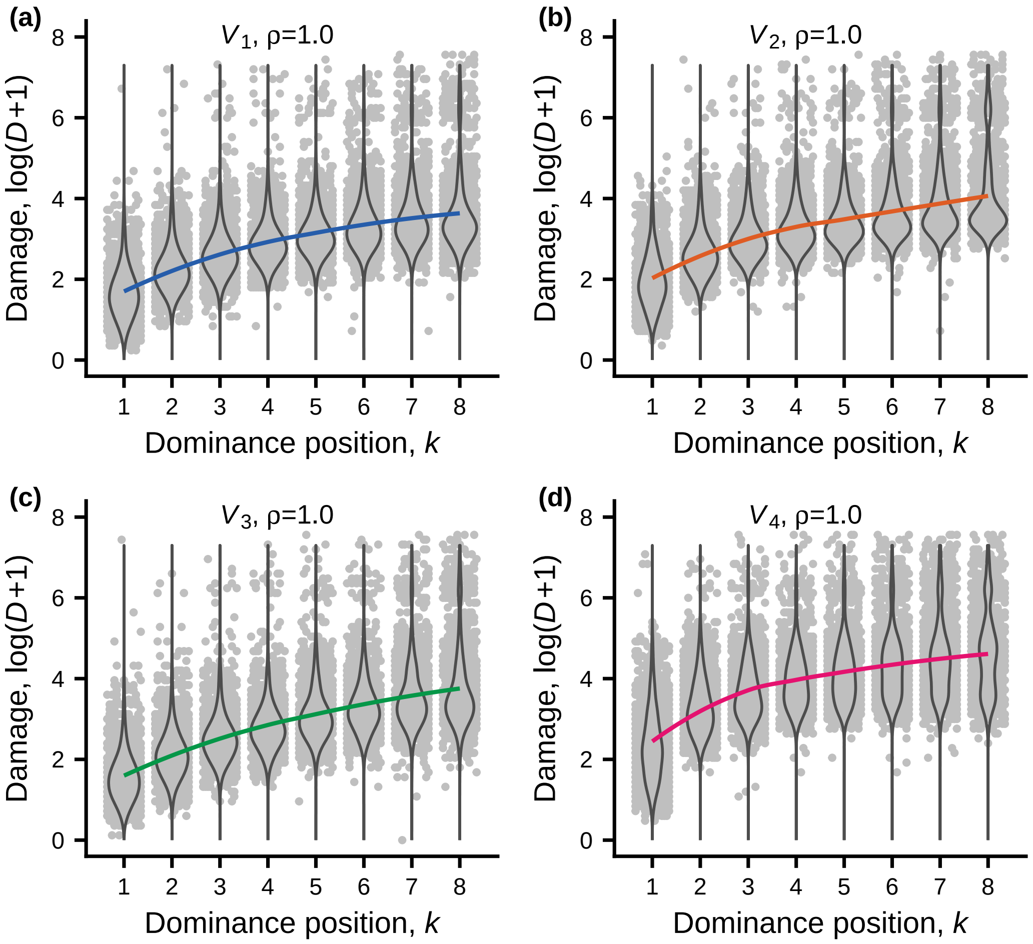

Figure S5: Simulated data (light grey points), density violins, and colour coded fitted curves of log-transformed total damage for the cases in Fig. 3b, 3d with $\rho = 1$. The x-values of the points are jittered for visibility.

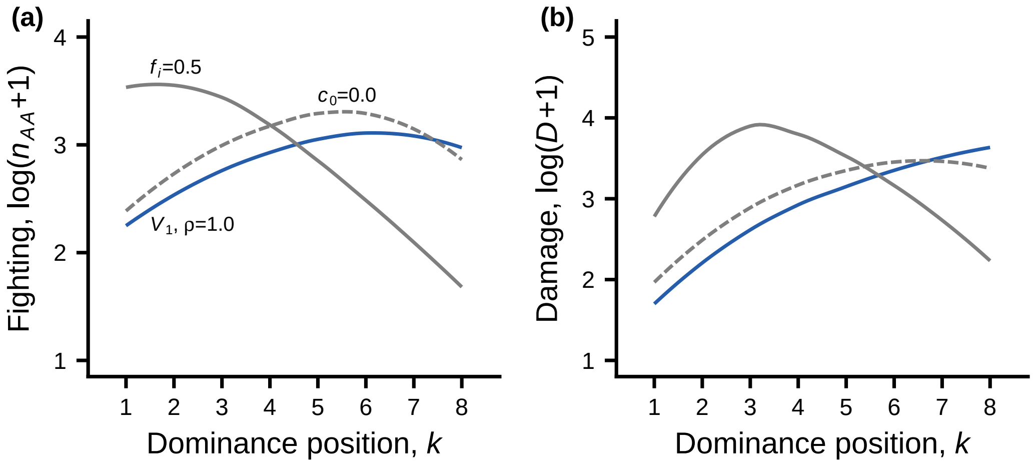

Figure S6: Illustration of the (non-evolutionary) effect of increasing the generalisation factor (solid grey curve) and eliminating the cost of loss of effective fighting ability (dashed grey curve), for the case of $V_1, \rho = 1$. The blue curves show the fitted log-transformed total number of AA rounds (fighting rounds) and total damage as functions of dominance position k, from Fig. 3a, 3b. The solid grey curve shows the effect of changing the generalisation factor from its evolved value of $f_i = 0.028$ (case 1 in Table S1) to the much higher value of $f_i = 0.5$. The dashed grey curves shows the effect of instead changing the nature of fighting damage, by eliminating the loss of effective fighting ability (i.e. putting $c_0 = 0$). Note that these changes are imposed on the situation of case 1 in Table S1, and do not represent evolutionary changes to different conditions.

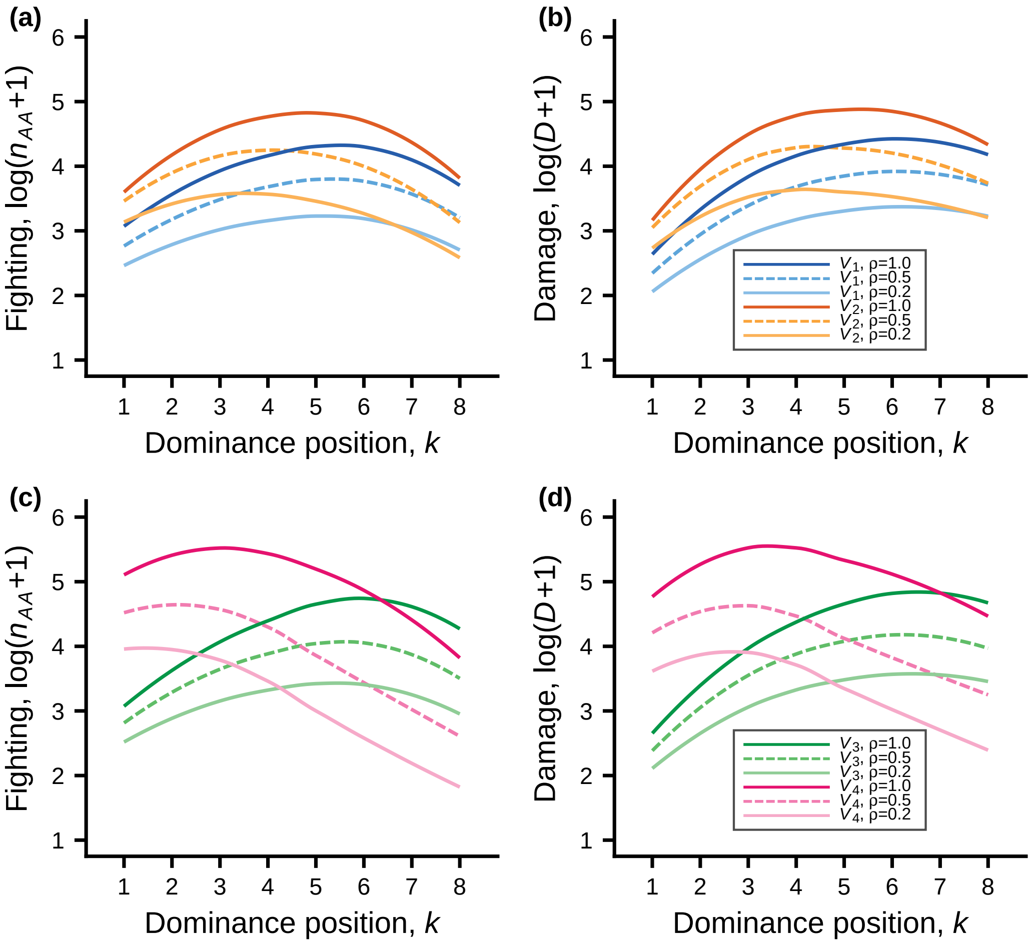

Figure S7: Fitted curves (loess fits) for log-transformed total number of AA rounds (fighting rounds) and total damage as functions of dominance position k, for the 12 evolutionary simulations in Table S2 (the learning parameters are given by the mean values in the table), with cases 13 to 18 in (a) and (b), and cases 19 to 24 in (c) and (d). For each case, 2000 groups of 8 individuals were simulated. The legends in panels (b) and (d) indicate the different cases, with colour coding as in Fig. 1c. Note that the difference between this figure and Fig. 3 is that here the traits have evolved in a situation where mortality is the only cost of fighting.
